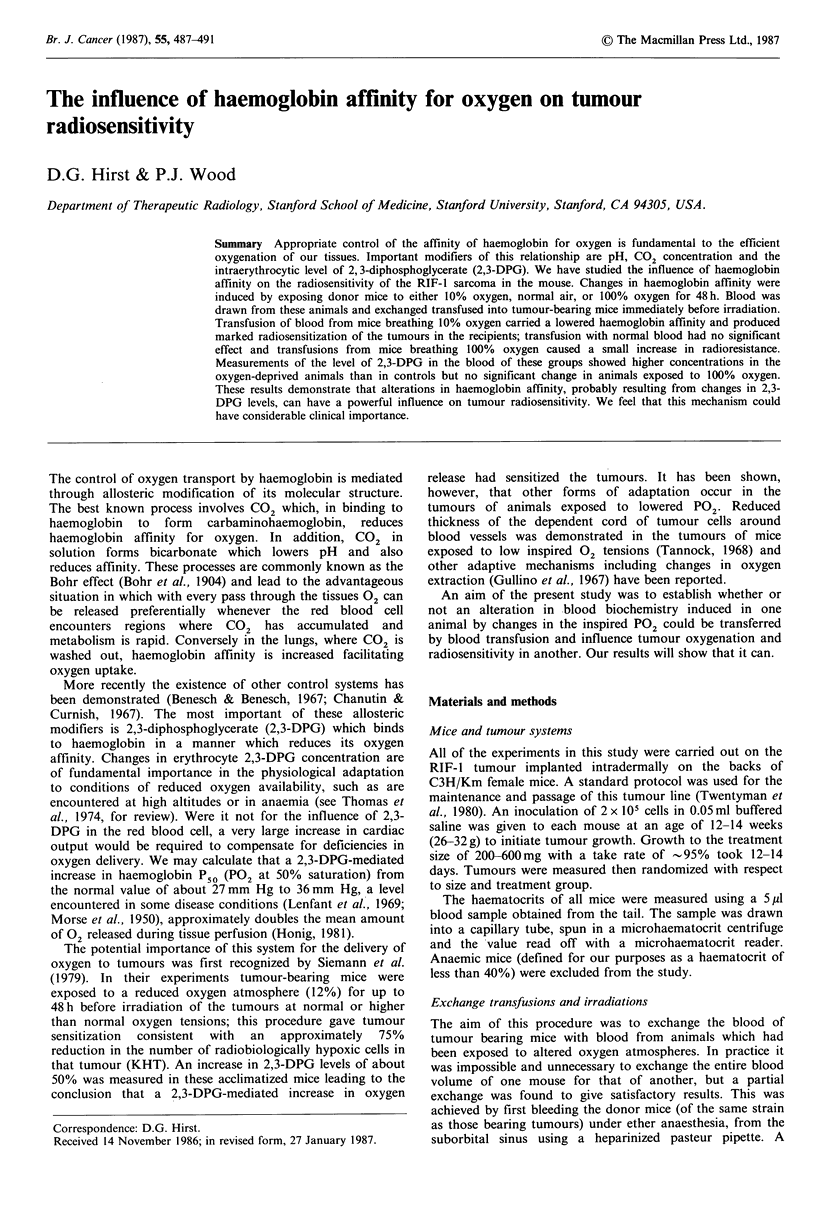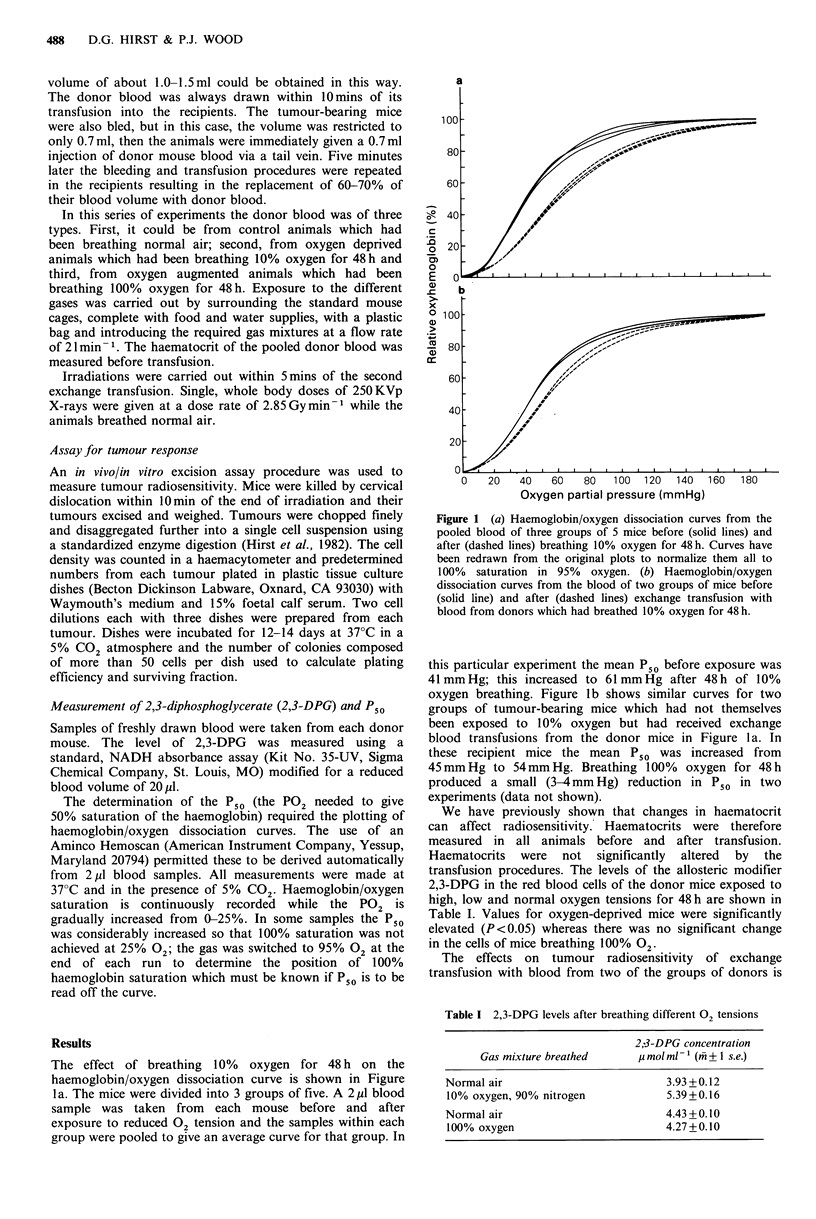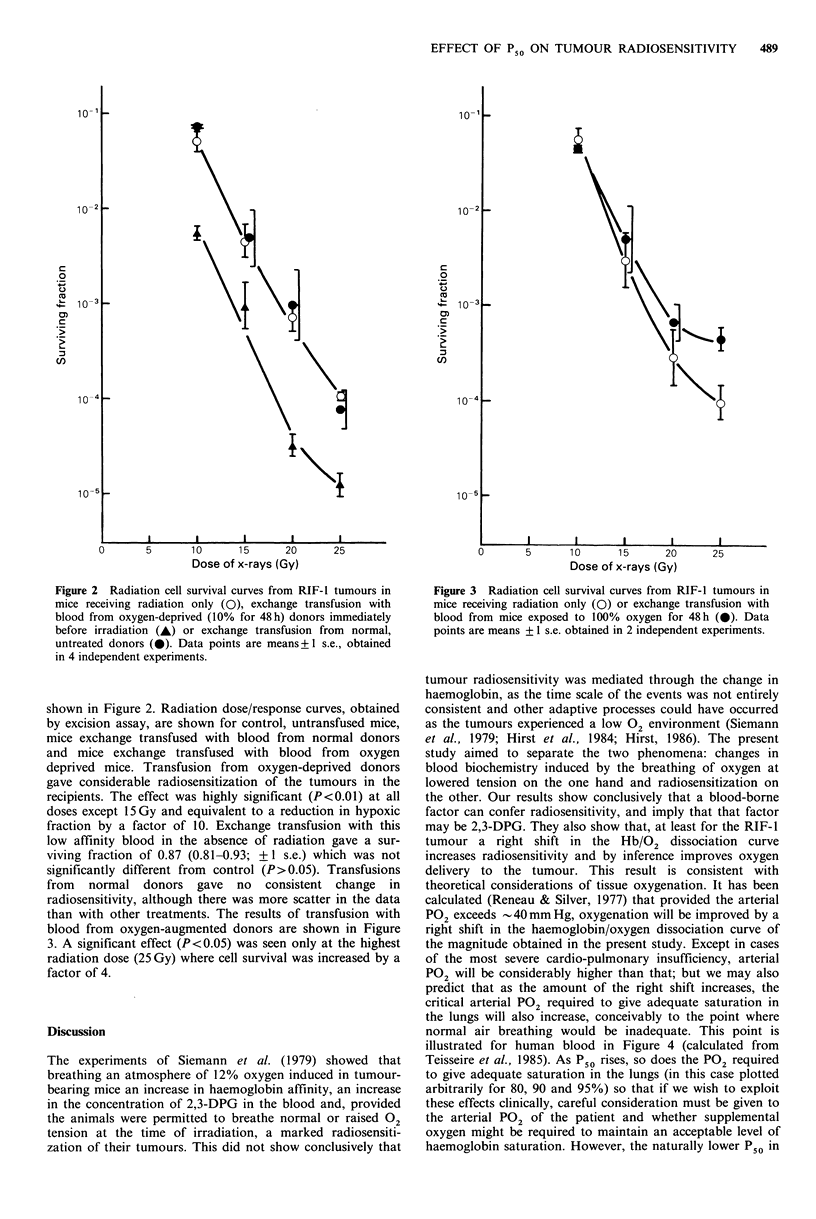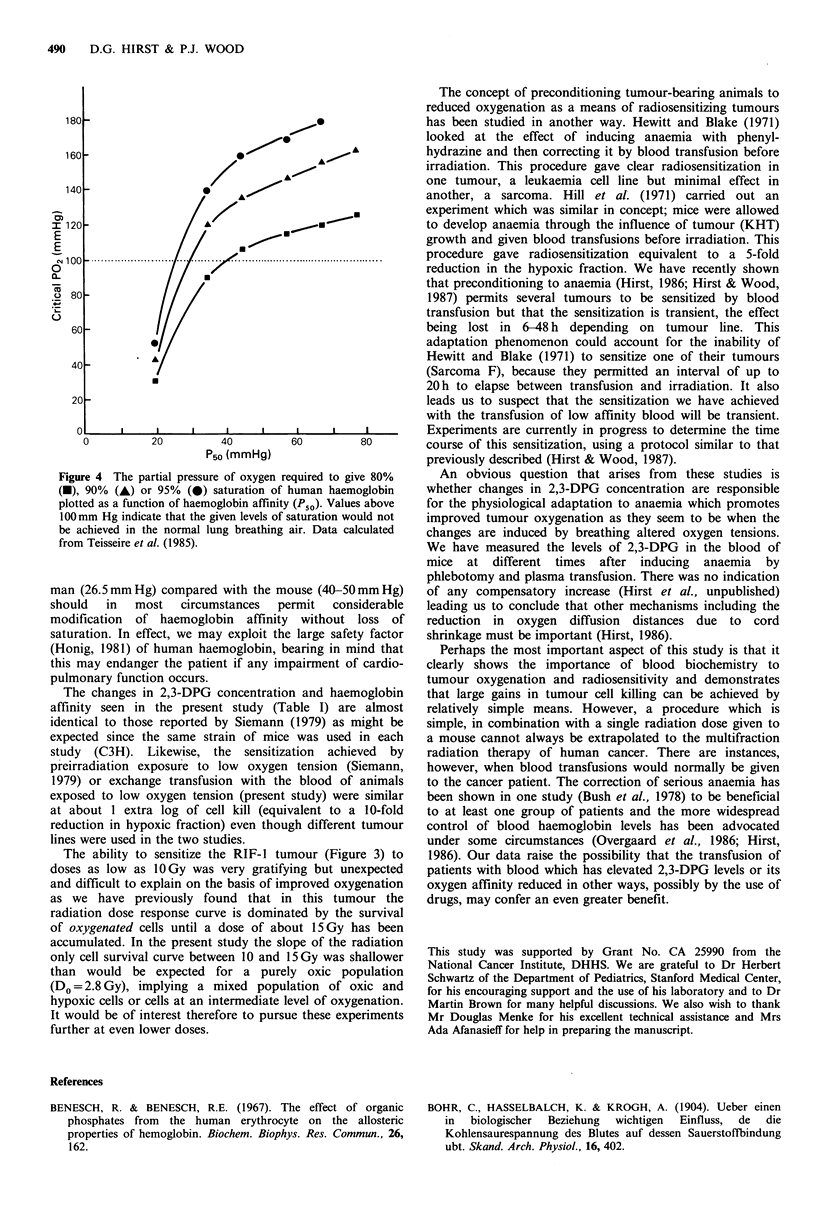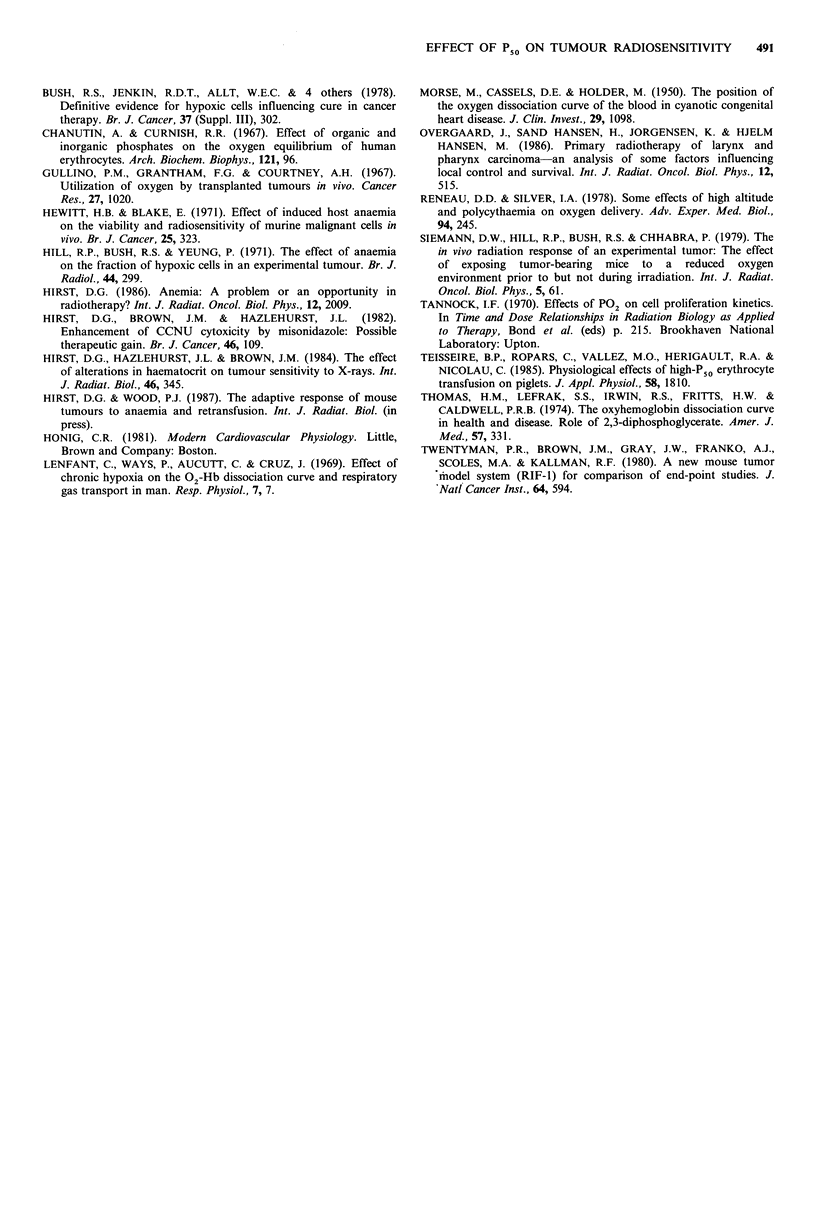# The influence of haemoglobin affinity for oxygen on tumour radiosensitivity.

**DOI:** 10.1038/bjc.1987.99

**Published:** 1987-05

**Authors:** D. G. Hirst, P. J. Wood

## Abstract

Appropriate control of the affinity of haemoglobin for oxygen is fundamental to the efficient oxygenation of our tissues. Important modifiers of this relationship are pH, CO2 concentration and the intraerythrocytic level of 2,3-diphosphoglycerate (2,3-DPG). We have studied the influence of haemoglobin affinity on the radiosensitivity of the RIF-1 sarcoma in the mouse. Changes in haemoglobin affinity were induced by exposing donor mice to either 10% oxygen, normal air, or 100% oxygen for 48 h. Blood was drawn from these animals and exchanged transfused into tumour-bearing mice immediately before irradiation. Transfusion of blood from mice breathing 10% oxygen carried a lowered haemoglobin affinity and produced marked radiosensitization of the tumours in the recipients; transfusion with normal blood had no significant effect and transfusions from mice breathing 100% oxygen caused a small increase in radioresistance. Measurements of the level of 2,3-DPG in the blood of these groups showed higher concentrations in the oxygen-deprived animals than in controls but no significant change in animals exposed to 100% oxygen. These results demonstrate that alterations in haemoglobin affinity, probably resulting from changes in 2,3-DPG levels, can have a powerful influence on tumour radiosensitivity. We feel that this mechanism could have considerable clinical importance.


Br. J. Cancer (1987), 55, 487–491 © The Macmillan Press Ltd., 1987

### The influence of haemoglobin affinity for oxygen on tumour radiosensitivity

D.G. Hirst & P.J. Wood

*Department of Therapeutic Radiology, Stanford School of Medicine, Stanford University, Stanford, CA 94305, USA.*

**Summary** Appropriate control of the affinity of haemoglobin for oxygen is fundamental to the efficient oxygenation of our tissues. Important modifiers of this relationship are pH, $CO_2$ concentration and the intraerythrocytic level of 2,3-diphosphoglycerate (2,3-DPG). We have studied the influence of haemoglobin affinity on the radiosensitivity of the RIF-1 sarcoma in the mouse. Changes in haemoglobin affinity were induced by exposing donor mice to either 10% oxygen, normal air, or 100% oxygen for 48 h. Blood was drawn from these animals and exchanged transfused into tumour-bearing mice immediately before irradiation. Transfusion of blood from mice breathing 10% oxygen carried a lowered haemoglobin affinity and produced marked radiosensitization of the tumours in the recipients; transfusion with normal blood had no significant effect and transfusions from mice breathing 100% oxygen caused a small increase in radioresistance. Measurements of the level of 2,3-DPG in the blood of these groups showed higher concentrations in the oxygen-deprived animals than in controls but no significant change in animals exposed to 100% oxygen. These results demonstrate that alterations in haemoglobin affinity, probably resulting from changes in 2,3-DPG levels, can have a powerful influence on tumour radiosensitivity. We feel that this mechanism could have considerable clinical importance.

The control of oxygen transport by haemoglobin is mediated through allosteric modification of its molecular structure. The best known process involves $CO_2$ which, in binding to haemoglobin to form carbaminohaemoglobin, reduces haemoglobin affinity for oxygen. In addition, $CO_2$ in solution forms bicarbonate which lowers pH and also reduces affinity. These processes are commonly known as the Bohr effect (Bohr *et al.*, 1904) and lead to the advantageous situation in which with every pass through the tissues $O_2$ can be released preferentially whenever the red blood cell encounters regions where $CO_2$ has accumulated and metabolism is rapid. Conversely in the lungs, where $CO_2$ is washed out, haemoglobin affinity is increased facilitating oxygen uptake.

More recently the existence of other control systems has been demonstrated (Benesch & Benesch, 1967; Chanutin & Curnish, 1967). The most important of these allosteric modifiers is 2,3-diphosphoglycerate (2,3-DPG) which binds to haemoglobin in a manner which reduces its oxygen affinity. Changes in erythrocyte 2,3-DPG concentration are of fundamental importance in the physiological adaptation to conditions of reduced oxygen availability, such as are encountered at high altitudes or in anaemia (see Thomas *et al.*, 1974, for review). Were it not for the influence of 2,3-DPG in the red blood cell, a very large increase in cardiac output would be required to compensate for deficiencies in oxygen delivery. We may calculate that a 2,3-DPG-mediated increase in haemoglobin $P_{50}$ ($PO_2$ at 50% saturation) from the normal value of about 27 mm Hg to 36 mm Hg, a level encountered in some disease conditions (Lenfant *et al.*, 1969; Morse *et al.*, 1950), approximately doubles the mean amount of $O_2$ released during tissue perfusion (Honig, 1981).

The potential importance of this system for the delivery of oxygen to tumours was first recognized by Siemann *et al.* (1979). In their experiments tumour-bearing mice were exposed to a reduced oxygen atmosphere (12%) for up to 48 h before irradiation of the tumours at normal or higher than normal oxygen tensions; this procedure gave tumour sensitization consistent with an approximately 75% reduction in the number of radiobiologically hypoxic cells in that tumour (KHT). An increase in 2,3-DPG levels of about 50% was measured in these acclimatized mice leading to the conclusion that a 2,3-DPG-mediated increase in oxygen release had sensitized the tumours. It has been shown, however, that other forms of adaptation occur in the tumours of animals exposed to lowered $PO_2$. Reduced thickness of the dependent cord of tumour cells around blood vessels was demonstrated in the tumours of mice exposed to low inspired $O_2$ tensions (Tannock, 1968) and other adaptive mechanisms including changes in oxygen extraction (Gullino *et al.*, 1967) have been reported.

An aim of the present study was to establish whether or not an alteration in blood biochemistry induced in one animal by changes in the inspired $PO_2$ could be transferred by blood transfusion and influence tumour oxygenation and radiosensitivity in another. Our results will show that it can.

### Materials and methods

### *Mice and tumour systems*

All of the experiments in this study were carried out on the RIF-1 tumour implanted intradermally on the backs of C3H/Km female mice. A standard protocol was used for the maintenance and passage of this tumour line (Twentyman *et al.*, 1980). An inoculation of $2 \times 10^5$ cells in 0.05 ml buffered saline was given to each mouse at an age of 12–14 weeks (26–32 g) to initiate tumour growth. Growth to the treatment size of 200–600 mg with a take rate of ~95% took 12–14 days. Tumours were measured then randomized with respect to size and treatment group.

The haematocrits of all mice were measured using a 5 $\mu$l blood sample obtained from the tail. The sample was drawn into a capillary tube, spun in a microhaematocrit centrifuge and the value read off with a microhaematocrit reader. Anaemic mice (defined for our purposes as a haematocrit of less than 40%) were excluded from the study.

### *Exchange transfusions and irradiations*

The aim of this procedure was to exchange the blood of tumour bearing mice with blood from animals which had been exposed to altered oxygen atmospheres. In practice it was impossible and unnecessary to exchange the entire blood volume of one mouse for that of another, but a partial exchange was found to give satisfactory results. This was achieved by first bleeding the donor mice (of the same strain as those bearing tumours) under ether anaesthesia, from the suborbital sinus using a heparinized pasteur pipette. A

Correspondence: D.G. Hirst.
Received 14 November 1986; in revised form, 27 January 1987.

volume of about 1.0–1.5 ml could be obtained in this way. The donor blood was always drawn within 10 mins of its transfusion into the recipients. The tumour-bearing mice were also bled, but in this case, the volume was restricted to only 0.7 ml, then the animals were immediately given a 0.7 ml injection of donor mouse blood via a tail vein. Five minutes later the bleeding and transfusion procedures were repeated in the recipients resulting in the replacement of 60–70% of their blood volume with donor blood.

In this series of experiments the donor blood was of three types. First, it could be from control animals which had been breathing normal air; second, from oxygen deprived animals which had been breathing 10% oxygen for 48 h and third, from oxygen augmented animals which had been breathing 100% oxygen for 48 h. Exposure to the different gases was carried out by surrounding the standard mouse cages, complete with food and water supplies, with a plastic bag and introducing the required gas mixtures at a flow rate of $2 l min^{-1}$. The haematocrit of the pooled donor blood was measured before transfusion.

Irradiations were carried out within 5 mins of the second exchange transfusion. Single, whole body doses of 250 KVp X-rays were given at a dose rate of $2.85 Gy min^{-1}$ while the animals breathed normal air.

### *Assay for tumour response*

An *in vivo/in vitro* excision assay procedure was used to measure tumour radiosensitivity. Mice were killed by cervical dislocation within 10 min of the end of irradiation and their tumours excised and weighed. Tumours were chopped finely and disaggregated further into a single cell suspension using a standardized enzyme digestion (Hirst *et al.*, 1982). The cell density was counted in a haemacytometer and predetermined numbers from each tumour plated in plastic tissue culture dishes (Becton Dickinson Labware, Oxnard, CA 93030) with Waymouth's medium and 15% foetal calf serum. Two cell dilutions each with three dishes were prepared from each tumour. Dishes were incubated for 12–14 days at 37°C in a 5% $CO_2$ atmosphere and the number of colonies composed of more than 50 cells per dish used to calculate plating efficiency and surviving fraction.

### *Measurement of 2,3-diphosphoglycerate (2,3-DPG) and $P_{50}$*

Samples of freshly drawn blood were taken from each donor mouse. The level of 2,3-DPG was measured using a standard, NADH absorbance assay (Kit No. 35-UV, Sigma Chemical Company, St. Louis, MO) modified for a reduced blood volume of 20 $\mu$l.

The determination of the $P_{50}$ (the $PO_2$ needed to give 50% saturation of the haemoglobin) required the plotting of haemoglobin/oxygen dissociation curves. The use of an Aminco Hemoscan (American Instrument Company, Yessup, Maryland 20794) permitted these to be derived automatically from 2 $\mu$l blood samples. All measurements were made at 37°C and in the presence of 5% $CO_2$. Haemoglobin/oxygen saturation is continuously recorded while the $PO_2$ is gradually increased from 0–25%. In some samples the $P_{50}$ was considerably increased so that 100% saturation was not achieved at 25% $O_2$; the gas was switched to 95% $O_2$ at the end of each run to determine the position of 100% haemoglobin saturation which must be known if $P_{50}$ is to be read off the curve.

### Results

The effect of breathing 10% oxygen for 48 h on the haemoglobin/oxygen dissociation curve is shown in Figure 1a. The mice were divided into 3 groups of five. A 2 $\mu$l blood sample was taken from each mouse before and after exposure to reduced $O_2$ tension and the samples within each group were pooled to give an average curve for that group. In this particular experiment the mean $P_{50}$ before exposure was 41 mmHg; this increased to 61 mmHg after 48 h of 10% oxygen breathing. Figure 1b shows similar curves for two groups of tumour-bearing mice which had not themselves been exposed to 10% oxygen but had received exchange blood transfusions from the donor mice in Figure 1a. In these recipient mice the mean $P_{50}$ was increased from 45 mmHg to 54 mmHg. Breathing 100% oxygen for 48 h produced a small (3–4 mmHg) reduction in $P_{50}$ in two experiments (data not shown).

**Figure 1** (*a*) Haemoglobin/oxygen dissociation curves from the pooled blood of three groups of 5 mice before (solid lines) and after (dashed lines) breathing 10% oxygen for 48 h. Curves have been redrawn from the original plots to normalize them all to 100% saturation in 95% oxygen. (*b*) Haemoglobin/oxygen dissociation curves from the blood of two groups of mice before (solid line) and after (dashed lines) exchange transfusion with blood from donors which had breathed 10% oxygen for 48 h.

We have previously shown that changes in haematocrit can affect radiosensitivity. Haematocrits were therefore measured in all animals before and after transfusion. Haematocrits were not significantly altered by the transfusion procedures. The levels of the allosteric modifier 2,3-DPG in the red blood cells of the donor mice exposed to high, low and normal oxygen tensions for 48 h are shown in Table I. Values for oxygen-deprived mice were significantly elevated ($P<0.05$) whereas there was no significant change in the cells of mice breathing 100% $O_2$.

The effects on tumour radiosensitivity of exchange transfusion with blood from two of the groups of donors is

**Table I** 2,3-DPG levels after breathing different $O_2$ tensions

| *Gas mixture breathed* | *2,3-DPG concentration* $\mu mol\, ml^{-1}$ ($\bar{m} \pm 1$ *s.e.*) |
|---|---|
| Normal air | $3.93 \pm 0.12$ |
| 10% oxygen, 90% nitrogen | $5.39 \pm 0.16$ |
| Normal air | $4.43 \pm 0.10$ |
| 100% oxygen | $4.27 \pm 0.10$ |

**Figure 2** Radiation cell survival curves from RIF-1 tumours in mice receiving radiation only (○), exchange transfusion with blood from oxygen-deprived (10% for 48 h) donors immediately before irradiation (▲) or exchange transfusion from normal, untreated donors (●). Data points are means ± 1 s.e., obtained in 4 independent experiments.

**Figure 3** Radiation cell survival curves from RIF-1 tumours in mice receiving radiation only (○) or exchange transfusion with blood from mice exposed to 100% oxygen for 48 h (●). Data points are means ± 1 s.e. obtained in 2 independent experiments.

shown in Figure 2. Radiation dose/response curves, obtained by excision assay, are shown for control, untransfused mice, mice exchange transfused with blood from normal donors and mice exchange transfused with blood from oxygen deprived mice. Transfusion from oxygen-deprived donors gave considerable radiosensitization of the tumours in the recipients. The effect was highly significant ($P<0.01$) at all doses except 15 Gy and equivalent to a reduction in hypoxic fraction by a factor of 10. Exchange transfusion with this low affinity blood in the absence of radiation gave a surviving fraction of 0.87 (0.81–0.93; ±1 s.e.) which was not significantly different from control ($P>0.05$). Transfusions from normal donors gave no consistent change in radiosensitivity, although there was more scatter in the data than with other treatments. The results of transfusion with blood from oxygen-augmented donors are shown in Figure 3. A significant effect ($P<0.05$) was seen only at the highest radiation dose (25 Gy) where cell survival was increased by a factor of 4.

### Discussion

The experiments of Siemann *et al.* (1979) showed that breathing an atmosphere of 12% oxygen induced in tumour-bearing mice an increase in haemoglobin affinity, an increase in the concentration of 2,3-DPG in the blood and, provided the animals were permitted to breathe normal or raised $O_2$ tension at the time of irradiation, a marked radiosensitization of their tumours. This did not show conclusively that tumour radiosensitivity was mediated through the change in haemoglobin, as the time scale of the events was not entirely consistent and other adaptive processes could have occurred as the tumours experienced a low $O_2$ environment (Siemann *et al.*, 1979; Hirst *et al.*, 1984; Hirst, 1986). The present study aimed to separate the two phenomena: changes in blood biochemistry induced by the breathing of oxygen at lowered tension on the one hand and radiosensitization on the other. Our results show conclusively that a blood-borne factor can confer radiosensitivity, and imply that that factor may be 2,3-DPG. They also show that, at least for the RIF-1 tumour a right shift in the $Hb/O_2$ dissociation curve increases radiosensitivity and by inference improves oxygen delivery to the tumour. This result is consistent with theoretical considerations of tissue oxygenation. It has been calculated (Reneau & Silver, 1977) that provided the arterial $PO_2$ exceeds ~40 mm Hg, oxygenation will be improved by a right shift in the haemoglobin/oxygen dissociation curve of the magnitude obtained in the present study. Except in cases of the most severe cardio-pulmonary insufficiency, arterial $PO_2$ will be considerably higher than that; but we may also predict that as the amount of the right shift increases, the critical arterial $PO_2$ required to give adequate saturation in the lungs will also increase, conceivably to the point where normal air breathing would be inadequate. This point is illustrated for human blood in Figure 4 (calculated from Teisseire *et al.*, 1985). As $P_{50}$ rises, so does the $PO_2$ required to give adequate saturation in the lungs (in this case plotted arbitrarily for 80, 90 and 95%) so that if we wish to exploit these effects clinically, careful consideration must be given to the arterial $PO_2$ of the patient and whether supplemental oxygen might be required to maintain an acceptable level of haemoglobin saturation. However, the naturally lower $P_{50}$ in

**Figure 4** The partial pressure of oxygen required to give 80% (■), 90% (▲) or 95% (●) saturation of human haemoglobin plotted as a function of haemoglobin affinity ($P_{50}$). Values above 100 mm Hg indicate that the given levels of saturation would not be achieved in the normal lung breathing air. Data calculated from Teisseire *et al.* (1985).

man (26.5 mm Hg) compared with the mouse (40–50 mm Hg) should in most circumstances permit considerable modification of haemoglobin affinity without loss of saturation. In effect, we may exploit the large safety factor (Honig, 1981) of human haemoglobin, bearing in mind that this may endanger the patient if any impairment of cardiopulmonary function occurs.

The changes in 2,3-DPG concentration and haemoglobin affinity seen in the present study (Table I) are almost identical to those reported by Siemann (1979) as might be expected since the same strain of mice was used in each study (C3H). Likewise, the sensitization achieved by preirradiation exposure to low oxygen tension (Siemann, 1979) or exchange transfusion with the blood of animals exposed to low oxygen tension (present study) were similar at about 1 extra log of cell kill (equivalent to a 10-fold reduction in hypoxic fraction) even though different tumour lines were used in the two studies.

The ability to sensitize the RIF-1 tumour (Figure 3) to doses as low as 10 Gy was very gratifying but unexpected and difficult to explain on the basis of improved oxygenation as we have previously found that in this tumour the radiation dose response curve is dominated by the survival of *oxygenated* cells until a dose of about 15 Gy has been accumulated. In the present study the slope of the radiation only cell survival curve between 10 and 15 Gy was shallower than would be expected for a purely oxic population ($D_0 = 2.8$ Gy), implying a mixed population of oxic and hypoxic cells or cells at an intermediate level of oxygenation. It would be of interest therefore to pursue these experiments further at even lower doses.

The concept of preconditioning tumour-bearing animals to reduced oxygenation as a means of radiosensitizing tumours has been studied in another way. Hewitt and Blake (1971) looked at the effect of inducing anaemia with phenylhydrazine and then correcting it by blood transfusion before irradiation. This procedure gave clear radiosensitization in one tumour, a leukaemia cell line but minimal effect in another, a sarcoma. Hill *et al.* (1971) carried out an experiment which was similar in concept; mice were allowed to develop anaemia through the influence of tumour (KHT) growth and given blood transfusions before irradiation. This procedure gave radiosensitization equivalent to a 5-fold reduction in the hypoxic fraction. We have recently shown that preconditioning to anaemia (Hirst, 1986; Hirst & Wood, 1987) permits several tumours to be sensitized by blood transfusion but that the sensitization is transient, the effect being lost in 6–48 h depending on tumour line. This adaptation phenomenon could account for the inability of Hewitt and Blake (1971) to sensitize one of their tumours (Sarcoma F), because they permitted an interval of up to 20 h to elapse between transfusion and irradiation. It also leads us to suspect that the sensitization we have achieved with the transfusion of low affinity blood will be transient. Experiments are currently in progress to determine the time course of this sensitization, using a protocol similar to that previously described (Hirst & Wood, 1987).

An obvious question that arises from these studies is whether changes in 2,3-DPG concentration are responsible for the physiological adaptation to anaemia which promotes improved tumour oxygenation as they seem to be when the changes are induced by breathing altered oxygen tensions. We have measured the levels of 2,3-DPG in the blood of mice at different times after inducing anaemia by phlebotomy and plasma transfusion. There was no indication of any compensatory increase (Hirst *et al.*, unpublished) leading us to conclude that other mechanisms including the reduction in oxygen diffusion distances due to cord shrinkage must be important (Hirst, 1986).

Perhaps the most important aspect of this study is that it clearly shows the importance of blood biochemistry to tumour oxygenation and radiosensitivity and demonstrates that large gains in tumour cell killing can be achieved by relatively simple means. However, a procedure which is simple, in combination with a single radiation dose given to a mouse cannot always be extrapolated to the multifraction radiation therapy of human cancer. There are instances, however, when blood transfusions would normally be given to the cancer patient. The correction of serious anaemia has been shown in one study (Bush *et al.*, 1978) to be beneficial to at least one group of patients and the more widespread control of blood haemoglobin levels has been advocated under some circumstances (Overgaard *et al.*, 1986; Hirst, 1986). Our data raise the possibility that the transfusion of patients with blood which has elevated 2,3-DPG levels or its oxygen affinity reduced in other ways, possibly by the use of drugs, may confer an even greater benefit.

This study was supported by Grant No. CA 25990 from the National Cancer Institute, DHHS. We are grateful to Dr Herbert Schwartz of the Department of Pediatrics, Stanford Medical Center, for his encouraging support and the use of his laboratory and to Dr Martin Brown for many helpful discussions. We also wish to thank Mr Douglas Menke for his excellent technical assistance and Mrs Ada Afanasieff for help in preparing the manuscript.

### References

BENESCH, R. & BENESCH, R.E. (1967). The effect of organic phosphates from the human erythrocyte on the allosteric properties of hemoglobin. *Biochem. Biophys. Res. Commun.*, **26**, 162.

BOHR, C., HASSELBALCH, K. & KROGH, A. (1904). Ueber einen in biologischer Beziehung wichtigen Einfluss, de die Kohlensaurespannung des Blutes auf dessen Sauerstoffbindung ubt. *Skand. Arch. Physiol.*, **16**, 402.

BUSH, R.S., JENKIN, R.D.T., ALLT, W.E.C. & 4 others (1978). Definitive evidence for hypoxic cells influencing cure in cancer therapy. *Br. J. Cancer*, **37** (Suppl. III), 302.

CHANUTIN, A. & CURNISH, R.R. (1967). Effect of organic and inorganic phosphates on the oxygen equilibrium of human erythrocytes. *Arch. Biochem. Biophys.*, **121**, 96.

GULLINO, P.M., GRANTHAM, F.G. & COURTNEY, A.H. (1967). Utilization of oxygen by transplanted tumours *in vivo*. *Cancer Res.*, **27**, 1020.

HEWITT, H.B. & BLAKE, E. (1971). Effect of induced host anaemia on the viability and radiosensitivity of murine malignant cells *in vivo*. *Br. J. Cancer*, **25**, 323.

HILL, R.P., BUSH, R.S. & YEUNG, P. (1971). The effect of anaemia on the fraction of hypoxic cells in an experimental tumour. *Br. J. Radiol.*, **44**, 299.

HIRST, D.G. (1986). Anemia: A problem or an opportunity in radiotherapy? *Int. J. Radiat. Oncol. Biol. Phys.*, **12**, 2009.

HIRST, D.G., BROWN, J.M. & HAZLEHURST, J.L. (1982). Enhancement of CCNU cytoxicity by misonidazole: Possible therapeutic gain. *Br. J. Cancer*, **46**, 109.

HIRST, D.G., HAZLEHURST, J.L. & BROWN, J.M. (1984). The effect of alterations in haematocrit on tumour sensitivity to X-rays. *Int. J. Radiat. Biol.*, **46**, 345.

HIRST, D.G. & WOOD, P.J. (1987). The adaptive response of mouse tumours to anaemia and retransfusion. *Int. J. Radiat. Biol.* (in press).

HONIG, C.R. (1981). *Modern Cardiovascular Physiology*. Little, Brown and Company: Boston.

LENFANT, C., WAYS, P., AUCUTT, C. & CRUZ, J. (1969). Effect of chronic hypoxia on the $O_2$-Hb dissociation curve and respiratory gas transport in man. *Resp. Physiol.*, **7**, 7.

MORSE, M., CASSELS, D.E. & HOLDER, M. (1950). The position of the oxygen dissociation curve of the blood in cyanotic congenital heart disease. *J. Clin. Invest.*, **29**, 1098.

OVERGAARD, J., SAND HANSEN, H., JORGENSEN, K. & HJELM HANSEN, M. (1986). Primary radiotherapy of larynx and pharynx carcinoma—an analysis of some factors influencing local control and survival. *Int. J. Radiat. Oncol. Biol. Phys.*, **12**, 515.

RENEAU, D.D. & SILVER, I.A. (1978). Some effects of high altitude and polycythaemia on oxygen delivery. *Adv. Exper. Med. Biol.*, **94**, 245.

SIEMANN, D.W., HILL, R.P., BUSH, R.S. & CHHABRA, P. (1979). The *in vivo* radiation response of an experimental tumor: The effect of exposing tumor-bearing mice to a reduced oxygen environment prior to but not during irradiation. *Int. J. Radiat. Oncol. Biol. Phys.*, **5**, 61.

TANNOCK, I.F. (1970). Effects of $PO_2$ on cell proliferation kinetics. In *Time and Dose Relationships in Radiation Biology as Applied to Therapy*, Bond *et al.* (eds) p. 215. Brookhaven National Laboratory: Upton.

TEISSEIRE, B.P., ROPARS, C., VALLEZ, M.O., HERIGAULT, R.A. & NICOLAU, C. (1985). Physiological effects of high-$P_{50}$ erythrocyte transfusion on piglets. *J. Appl. Physiol.*, **58**, 1810.

THOMAS, H.M., LEFRAK, S.S., IRWIN, R.S., FRITTS, H.W. & CALDWELL, P.R.B. (1974). The oxyhemoglobin dissociation curve in health and disease. Role of 2,3-diphosphoglycerate. *Amer. J. Med.*, **57**, 331.

TWENTYMAN, P.R., BROWN, J.M., GRAY, J.W., FRANKO, A.J., SCOLES, M.A. & KALLMAN, R.F. (1980). A new mouse tumor model system (RIF-1) for comparison of end-point studies. *J. Natl Cancer Inst.*, **64**, 594.